# Cerebellar Influence on Motor Cortex Plasticity: Behavioral Implications for Parkinson’s Disease

**DOI:** 10.3389/fneur.2014.00068

**Published:** 2014-05-06

**Authors:** Asha Kishore, Sabine Meunier, Traian Popa

**Affiliations:** ^1^Department of Neurology, Comprehensive Care Centre for Movement Disorders, Sree Chitra Tirunal Institute for Medical Sciences and Technology, Kerala, India; ^2^Institut du Cerveau et de la Moelle epiniere (ICM), INSERM U1127, CNRS UMR 7225, Université Pierre et Marie Curie-Paris 6 UMR S975, Paris, France; ^3^Centre de Neuroimagerie de Recherche (CENIR), l’Institut du Cerveau et de la Moelle epiniere (ICM), Paris, France

**Keywords:** motor cortex plasticity, Parkinson disease, cerebellum, dopamine, basal ganglia

## Abstract

Normal motor behavior involves the creation of appropriate activity patterns across motor networks, enabling firing synchrony, synaptic integration, and normal functioning of these networks. Strong topography-specific connections among the basal ganglia, cerebellum, and their projections to overlapping areas in the motor cortices suggest that these networks could influence each other’s plastic responses and functions. The defective striatal signaling in Parkinson’s disease (PD) could therefore lead to abnormal oscillatory activity and aberrant plasticity at multiple levels within the interlinked motor networks. Normal striatal dopaminergic signaling and cerebellar sensory processing functions influence the scaling and topographic specificity of M1 plasticity. Both these functions are abnormal in PD and appear to contribute to the abnormal M1 plasticity. Defective motor map plasticity and topographic specificity within M1 could lead to incorrect muscle synergies, which could manifest as abnormal or undesired movements, and as abnormal motor learning in PD. We propose that the loss of M1 plasticity in PD reflects a loss of co-ordination among the basal ganglia, cerebellar, and cortical inputs which translates to an abnormal plasticity of motor maps within M1 and eventually to some of the motor signs of PD. The initial benefits of dopamine replacement therapy on M1 plasticity and motor signs are lost during the progressive course of disease. Levodopa-induced dyskinesias in patients with advanced PD is linked to a loss of M1 sensorimotor plasticity and the attenuation of dyskinesias by cerebellar inhibitory stimulation is associated with restoration of M1 plasticity. Complimentary interventions should target reestablishing physiological communication between the striatal and cerebellar circuits, and within striato-cerebellar loop. This may facilitate correct motor synergies and reduce abnormal movements in PD.

## Introduction

Motor cortex, basal ganglia, and cerebellum have unique architectures and synaptic mechanisms that allow specialized forms of information processing (1). Traditionally, motor cortex is considered to be specialized for unsupervised learning through Hebbian plasticity mechanisms (2). Basal ganglia are considered necessary for selection of wanted movements and inhibition of unwanted movements (3). They are specialized for reinforcement learning, based on reward signals encoded in dopaminergic fibers (4). The cerebellum is thought to fine tune movements in real time and to be specialized for supervised learning, based on error signals encoded in the climbing fibers (5). Such compartmentalized roles, though not clear-cut, were perhaps assigned to these structures because of the then-prevailing model that basal ganglia and cerebellum were distinct, parallel systems, each with reciprocal connections through the thalamus to multiple cortical areas (6). In recent years, direct bisynaptic connections between the motor areas of the dentate nucleus and striatum via thalamus were demonstrated in monkeys (7). Later, topographically organized connections that link the subthalamic nucleus and cerebellum through the pontine nuclei (8) were also identified in monkeys. The subthalamic nucleus projections to the motor area VIIB of cerebellum arise from the sensorimotor territory of subthalamic nucleus, which in turn, is under the influence of M1 and pre-motor areas. These well-organized, topography-specific connections among the motor and pre-motor cortices, basal ganglia, and cerebellum indicate that these structures may interact synergistically in humans. This could mean that the information processed by each of them may significantly influence the functioning of all related structures. If so, abnormal dopaminergic release as in Parkinson’s disease (PD), could affect macro- and micro-circuit functions in the striatum, cerebellum, and sensorimotor cortical areas. Consequently, the burden of symptoms in PD, both motor and non-motor, may result from dysfunctions within this large network. We review here the potential relation between striato-thalamo-cortical and cerebello-thalamo-cortical loops and M1 plasticity impairment in PD, and how their progressive imbalance could lead to continuously deteriorating manifestations.

## M1 Plasticity and Motor Learning

M1 is interconnected with the parietal lobe, pre-motor cortex, supplementary motor area, basal ganglia, and cerebellum. This strategic location within a distributed system (9) underlies M1’s pivotal role in motor performance and motor learning. The capacity of M1 to offer behavioral flexibility to motor functions is dependent on its ability to modify its local architecture and promote dynamic motor maps. M1 is organized as motor maps consisting of somatotopically arranged representations of muscle synergies. The muscle synergies can be represented by the weight of neural connections. The cortical areas in which the movement representations are embedded have strong interconnections (10) that are highly dynamic and capable of rapid reorganization (11). Task-specific modification in spatial and temporal organization of muscle synergies results in smooth and accurate movement sequences (12). Encoding of a novel movement sequence in motor cortex results in changes in the weight of connectivity, when sets of movements are performed together (13). The representation strength of a sequence is increased as movements are learned and this comes with an expansion of the cortical motor map encoding the specific movement or skill (14). Such organizational capability of the human M1 was demonstrated using transcranial magnetic stimulation techniques (15, 16). The plastic changes in cortical map are thought to occur through reorganization of cortical micro-circuitry and changes in synaptic efficacy (17). Changes in synaptic efficacy occur first through processes that involve long-term potentiation (LTP) and long-term depression (LTD), and then through changes in synaptic architecture. The pyramidal neurons in M1 have extensive networks of branches that establish horizontal connections within M1 (18). The synaptic efficacy of these interconnections can be enhanced or depressed through LTP and LTD, in response to appropriate activity patterns and contexts (11). M1 is also under the influence of other cortical areas (e.g., parietal, pre-motor) and subcortical structures, including the basal ganglia, cerebellum, and brain stem. The timing of cortico-cortical and subcortico-cortical inputs controls the buildup of LTP and LTD within M1. This translates into change in synaptic strength that facilitates only sensorimotor input from subcortical structures that are relevant to a specific context (11). With repetition, these reversible changes can lead to physical reorganization of intracortical connections. The integrity of motor maps and their topography in M1 are also influenced by neurochemical signals that change the cortical circuitry to encode the motor experience (19). Disrupting cortical circuitry by inhibition of protein synthesis or ischemic injury results in a loss of motor maps and degradation of skilled movement (20). These observations suggest that motor maps and their recruitment pattern required for performance of appropriate movements are based on a high level of synergy within M1, and between M1 and other structures.

## Cortico-Striatal Plasticity

Striatum is a major input station of glutamatergic projections from the motor cortices and the thalamus. It is also densely innervated by dopaminergic terminals arising from the *substantia nigra pars compacta*. The integration of informational flow within the striatum determines the final output to other basal ganglia structures. LTP and LTD are the key cellular substrates for motor control and learning subserved by the striatum (21). Induction of plasticity at the cortico-striatal synapses with the medium spiny neurons (MSNs) requires interaction between dopamine and NMDA receptors. NMDA receptor’s complex modification linked to dopamine D1 receptor activation eventually leads to postsynaptic insertion of AMPA receptors that underlies LTP (22). Both D1 and D2 receptor activation and metabotropic NMDA receptors are involved in LTD. Dopamine has a rapid, reversible action that can transiently alter synaptic integration and microcircuit function to enhance the transfer of specific types of information through the striatum. Additionally, dopamine also has a slow action that can induce persistent changes that outlast the dopaminergic signal and translate to long-term motor memory (23). The striatal MSNs are connected to the pallidal output neurons though the direct and indirect pathways. D1 receptor activation has excitatory effects on striatal MSNs in the direct-pathway while D2 receptor activation has inhibitory effects on MSNs in the indirect pathway (24). It has been proposed that activation of direct-pathway circuits facilitates or selects appropriate movements, while activity in the indirect pathway inhibits inappropriate movements (3, 25). Since the striatal release of dopamine can signal a “reward prediction error” (4, 26), basal ganglia could participate in motor learning through the selection of a motor routine by maintaining wanted and eliminating unwanted movements in a precise temporal sequence. Correctly performed actions would lead to micro self-rewarding results, which reinforce the choice of actions that led to the successful outcome. In course of time, the action sequences associate with each other, allowing the rapid selection of motor routines independent of reward values, thus becoming automatic (23). Therefore, basal ganglia can render movements more efficiently, by comparing the input from the motor cortex with the locally stored motor routines, and predict likely future actions. This type of learning is thought to occur through long-term changes in the strength of striatal synapses (27).

## Dopamine and M1 Plasticity

In rats, there is a large dopaminergic projection to M1 through the mesocortical system arising in the ventral tegmental area and the medial substantia nigra. Larger and similar motor cortical innervations are also reported in primates (28), which is not different from humans (29). In rats, mesocortical dopaminergic signaling is necessary for the intracortical and cortico-cortical connections of M1 to form LTP. Blocking dopamine D1 and D2 receptors reduce this ability of M1 (30). In humans, mesocortical projections from the ventral tegmental area to the prefrontal cortex and their role in motor function have been studied (30–32). Both dopamine D1 and D2 receptors are present in human M1 (18) and D2 receptor-blocking drugs can prevent LTP (33). The role of dopaminergic transmission in the ventral tegmental area to M1 projection described in humans (34), in M1 plasticity and motor function (both in health and PD) still need to be explored. Plasticity of human M1 is thought to be influenced by striatal dopaminergic system through the glutamatergic striato-thalamo-cortical pathway. This conclusion is supported by the observation that the lost M1 plasticity in PD recovers after exposure to dopaminergic drugs (35). However, cortical dopaminergic denervation by itself could cause the loss of M1 plasticity in PD and dopaminergic drugs could also act directly through the cortical dopamine receptors restore M1 plasticity in early stages of the disease.

## Cerebellar Plasticity

Cerebellum controls and co-ordinates complex movements and is important for adapting movements to changes in feedback. It receives sensory and motor information from descending cortical pathways and ascending peripheral pathways. It has also connections to the parietal, pre-motor, and frontal cortices. The two major excitatory afferents to cerebellum are the climbing fibers and mossy fiber–parallel fiber systems, both of which eventually converge on the Purkinje cells, which are the only efferent output from the cerebellar cortex. The exteroceptive and proprioceptive inputs from the spinal cord and the pontine input convey information from brain stem nuclei via mossy fibers to the granule cells. The axons of granule cells form the parallel fibers network. Climbing fibers originate in the inferior olive and directly relay to the Purkinje cells. Plastic changes in the strength of synapses relaying from the climbing and parallel fibers to the Purkinje cells are important in motor learning (36–40). Moreover, plasticity in both granule cell and Purkinje cell networks are required for motor learning and consolidation (41). In addition, there is inhibitory plasticity at the inhibitory interneuron-PC synapses as well intrinsic plasticity mechanisms within the cerebellum (42). There are several theories on the role of cerebellum in motor learning. It was considered that LTD of parallel fibers–Purkinje cell synapses, which in turn required simultaneous co-activation of parallel fibers and climbing fibers inputs to Purkinje cells, provided the cellular correlate of motor learning (36). More recent observations suggest that climbing fibers signaling has a more complex role. Plasticity of climbing fibers input can additionally fine tune complex spike-associated calcium signaling in Purkinje cells and bi-directionally adjust the plasticity of parallel fibers–Purkinje cells synapses (43). This suggests a role of climbing fibers as an error detector preceding motor learning, which signals the need for adjusting the gain of sensory inputs and/or motor output within the cerebellum (44). Based on the circuit architecture around the Purkinje cells, Penhune and Steele proposed that cerebellum participates in sensorimotor integration, error correction, and formation of internal models (45). In this context, internal models were defined as a set of input–output relations between motor commands and their sensory consequences, the input being the motor command and the output being the predicted sensory consequence of that action. Internal models subsequently allow comparison between predicted and actual consequences of a movement. This would enable gauging the movement error signal that guides learning. The authors suggest that internal models in the parallel fibers–Purkinje cells complexes may be modified based on the information about motor plans from motor cortex and on the error signals transmitted from the inferior olive. This can update the relationship between the command to move and the expected sensory consequence. Any disturbance in this cerebellar processing function may result in maladjusted information delivered to M1, leading to abnormal, undesired or ineffective movement sequences.

## Cerebellum and M1 Plasticity

The primary motor cortex is functionally linked specifically with cerebellar lobules V, VI, VIIB, and VIIIA, which are also implicated in motor learning (46, 47). Animal experiments (47–49) and human imaging studies showed that cerebellum is involved in sensory processing besides facilitating motor control and motor learning (50, 51). One view describes the cerebellum function as an adaptive filter (52). It was recently shown that cerebellum plays a very important role in scaling plasticity and influencing topographic specificity of the human M1 through modulation of peripheral sensory afferents (53). In healthy young adults, excitation or inhibition of the posterior cerebellar cortex (using theta-burst transcranial magnetic stimulation) preceding the induction of M1 plasticity, had bidirectional effects on M1 plasticity. Cerebellar cortical excitation led to a loss of response to a subsequent excitatory stimulation protocol pairing somatosensory stimulation paired to TMS applied precisely to target only one muscle representation within M1. In contrast, cerebellar cortical inhibition led to a prolonged plastic response of M1 to the paired-associative stimulation protocol along with a loss of topographic specificity, i.e., changes in both targeted and adjacent, non-targeted muscle representations. This suggested a highly discriminating role of cerebellar excitatory and inhibitory functional outputs to M1. These alterations in the response of M1 following cerebellar modulation were observed for PAS but not for theta-burst transcranial magnetic stimulation (which does not rely on sensory afferent input). This particular behavior highlights the dependence of cerebellar modulation of M1 plasticity on the sensory afferent input. Cerebellar cortical excitation could lead to an enhancement of the normal inhibition of dentate nucleus by the Purkinje cells. This would reduce the normal excitatory control of dentate nucleus on the afferent inflow to M1, probably at the thalamic or olivary nuclear level, thus blocking the sensorimotor-plasticity within M1 (53). The functional relevance of such cerebellar modulation of M1 plasticity could be to prevent the selection of unsuited or new motor programs from sources external to M1 and provide stability to motor maps. In contrast, cerebellar cortical inhibition could lead to disinhibition of dentate nucleus, thus facilitating afferent input to M1 and thereby providing a “controlled instability” of motor maps, which might enable updating the currently selected motor programs by facilitating the insertion of elements of a new motor program.

## Cortico-Striatal and M1 Plasticity in Parkinson’s Disease

**Figure 1:**
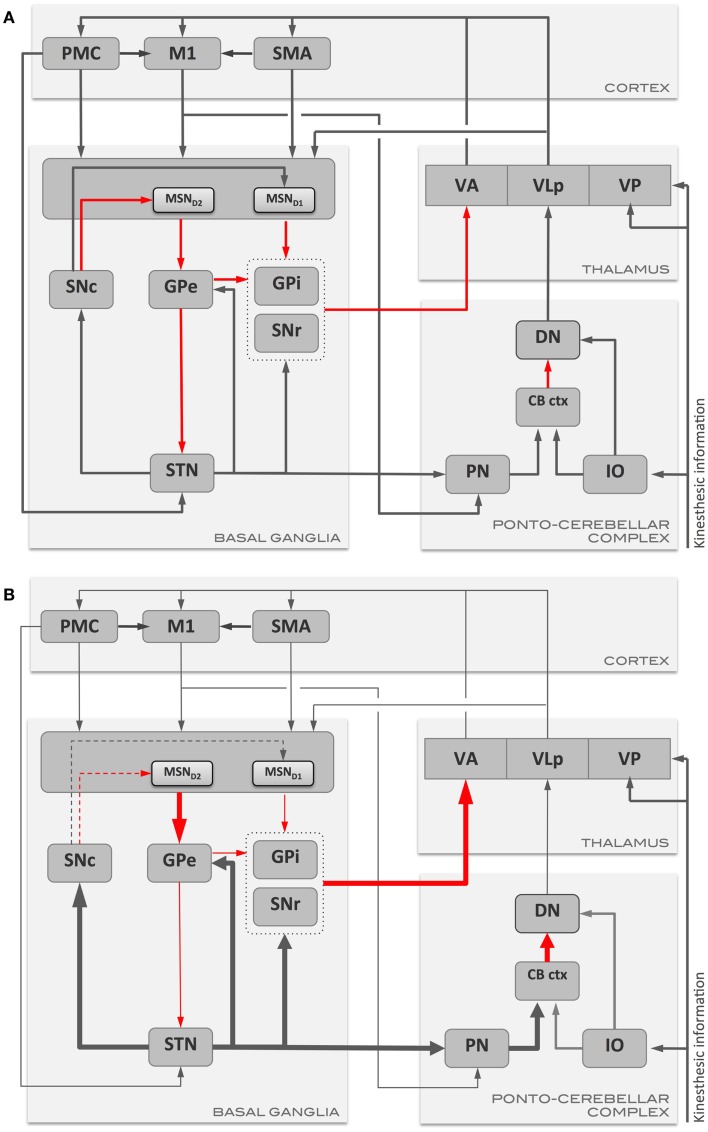
**Schematic representation of the main communication pathways between the motor areas, basal ganglia, and ponto-cerebellar complex in (A) health, and in (B) Parkinson’s disease**. Gray arrows represent excitatory pathways, and red arrows represent inhibitory pathways. Thickened arrows in **(B)** represent pathways with augmented output with respect to the normal condition **(A)**, while thinned arrows represent pathways with reduced output. CB ctx, cerebellar cortex; DN, dentate nucleus; GPe, globus pallidus externus; GPi, globus pallidus internus; IO, inferior olive; M1, primary motor cortex; MSN, medium spiny neurons (MSN_D1/D2_: MSN with D1 or D2 dopamine receptors); PMC, pre-motor cortex; PN, pontine nuclei; SMA, supplementary motor area; SNc, substantia nigra pars compacta; SNr, substantia nigra pars reticulata; STN, subthalamic nucleus; VA, ventro-anterior thalamic nucleus; VLp, ventro-lateral thalamic nucleus, pars posteriori; VP, ventro-posterior thalamic nucleus.

Parkinson’s disease is characterized by a massive loss of dopaminergic neurons in the midbrain (54) and degeneration of catecholaminergic neurons in other parts of the brainstem (55). Dopamine deficiency at the striatum results in loss of both LTP in the direct pathway and loss of LTD and its replacement by LTP in the indirect pathway (56). Striatal dopamine depletion also leads to enhanced indirect pathway output and decreased direct-pathway output (Figure 1). This results in a decrease in activity in GPe and increase in subthalamic nucleus and GPi both in experimental (57, 58) and human PD (59). As the indirect pathway normally inhibits unwanted movements, the loss of LTD and over-activity in this pathway could result in inhibition of wanted movements and a disruption of learned motor actions. With the loss of LTD, the MSNs in the indirect pathway are liable to increased entrainment to the oscillations in thalamus and cortex through their inputs to striatum (60). As the direct pathway activity normally selects appropriate movements, it’s under activity and loss of LTP could affect initiation and performance of appropriate movements in PD (56, 61). There are intrinsic difficulties in differentiating abnormal motor learning in the presence of abnormal motor performance. Even so, it has been shown that motor learning is abnormal in PD (62, 63). Beeler and colleagues proposed that motor learning may play a significant role in the symptoms of PD and that the long-duration response to chronic levodopa treatment may be a manifestation of rescued motor learning. This was based on observations in the aphakia mouse model that lacked 90% dopamine in the dorsal striatum resulting in impaired new motor learning skills but without motor deficits (64). l-DOPA rescued motor learning and cessation of treatment did not result in an immediate loss of the rescued motor learning skills.

l-DOPA-responsive, severe impairment of striatal plasticity was first demonstrated in animal models of PD (65). A similar impairment of LTP that responded to l-DOPA was also demonstrated in human *substantia nigra pars reticulata* (66) in PD patients tested during deep brain stimulation surgery. A decreased response of M1 to LTP- and LTD-inducing protocols was also documented in human M1 in the more affected hemisphere in most studies in PD patients (30, 67, 68). This has been interpreted to reflect an impairment of LTP/LTD mechanisms within M1. The defective plasticity in PD demonstrated at the output neurons of *substantia nigra pars reticulata* (69) and M1 (67–70) could be secondary to the abnormal signaling in the striatal circuits. However, the loss of direct effects of dopaminergic input from ventral tegmental area on M1 might contribute to the M1 plasticity alterations in PD (67). In *de novo* PD patients, the intrinsic, plasticity of M1 involving local circuits within M1 (as probed by TMS) was severely and symmetrically impaired in both hemispheres, even when there were only hemi-parkinsonian symptoms (67). The deficient plasticity did not improve with a single dose of l-DOPA even though motor signs of PD improved. Nevertheless, chronic dopamine treatment restored TMS-induced intrinsic plasticity within local M1 circuits in the stable phase of treatment in a manner akin to the long-duration response of motor signs. In the stage of established motor fluctuations and levodopa-induced dyskinesias (LIDs), M1 plasticity reverted to a severe impairment, indicating a loss of long-duration response of M1 plasticity (70). The sensorimotor, M1 plasticity (as probed by paired-associative stimulation protocol) was also impaired in *de novo* PD patients in the more affected hemisphere, while the less affected hemisphere showed preserved plasticity with loss of topographic specificity of the plastic effect (71). The latter effect was attributed to either the disease process or a compensatory adjustment to reduce the severity of motor signs. In patients with more advanced disease and experiencing motor fluctuations and dyskinesias, both the sensorimotor, plasticity (69) and the plasticity of M1 (70) were lost, and both forms of plastic responses were l-DOPA unresponsive. When the synaptic milieu is unstable and compensatory mechanisms have failed at the cortico-striatal terminals (72), excessive synaptic release of dopamine (73) can swamp the plasticity-inducing intracellular cascade of events by affecting the functioning of key enzymes (74). In animal models of PD with LIDs, besides alterations in synaptic plasticity, there were modifications in the trafficking and subunit composition of NMDA receptors that were attributed to non-physiological dopaminergic stimulation (21). It is likely that the effects of progressive neuro-degeneration and the detrimental effects of non-physiological dopamine replacement therapy affect neural signaling and plasticity mechanisms not only at the striatum but also at the multiple nodes in the interlinked motor circuits that ultimately influence M1 plasticity.

## Cerebellum and Parkinson’s Disease

There is mounting evidence that besides abnormal basal ganglia signaling, cerebellar dysfunction also occurs in PD (75). In animal experiments, the two independent ventral thalamic areas receiving basal ganglia and cerebellar input show decreased neuronal firing following dopamine depletion, indicative of both altered basal ganglia and cerebellar outputs in PD (76). Information about motor plans (delivered by mossy fibers from motor cortices via pontine nuclei) and the error signals (delivered via climbing fibers from inferior olive) are both coded by excitatory inputs that are processed at level of the cerebellar cortex and deep nuclei. It is conceivable that a disturbance in this cerebellar processing function could result in undesired and ineffective movement sequences, by impairing M1 plasticity. A recent study reported that in a chronic model of drug-naïve MPTP-treated parkinsonian monkeys, the level of dopaminergic neuronal loss in *substantia nigra pars compacta* correlated with a persistent hyper-excitation of the Purkinje cells (77). A similar state of cerebellar over excitation may exist in human PD and could potentially affect the information processing within the cerebellum. In support of the cerebellar hyper-excitation model of PD, a SPECT study found heightened activity of the cerebellum at rest in PD patients compared to controls when off anti-parkinsonian medication but not when the patients were on medication (78). Additionally, resting-state MRI showed that l-DOPA increases the functional connectivity between putamen, cerebellum, and brain stem (79). Functional MRI studies showed hyper-activation of the cerebellum in PD patients during simple motor tasks (79–81). Reciprocally, lesions in the paravermal cerebellum in mice increase D1 receptor levels in the contralateral striatum (82) suggesting that cerebellar cortical and nuclear projections modulate the D1 receptor expression of the striatal direct pathway. Though the neuroimaging evidence of cerebellar over-activity was earlier interpreted as compensatory, the discovery of bidirectional communications between the basal ganglia and cerebellar circuits (7, 8) raises the important question of whether the cerebellar hyper-activation is linked to the abnormal striatal signaling in human PD. It is plausible that the pathologically increased excitatory output from the subthalamic nucleus in PD (83) could propagate to the cerebellum and induce a chronic hyper-excited state. This would prevent any discrete excitatory input from being efficiently processed, thus interfering with the cerebellar tuning of M1 plasticity (84). DBS of subthalamic nucleus that improves the clinical signs of PD may actually also contribute to reduce cerebellar over-activity in PD patients (85).

In late-stage PD, the chronic abnormal excitatory drive from the subthalamic nucleus to the cerebellar cortex might induce physical synaptic reconfigurations that lock the cerebellar cortex in hyper-excited state. Dopamine replacement therapy could normalize the basal ganglia output without being necessarily followed by an immediate and effective reduction of the cerebellar cortex excitation. This would result in a conflict between the normalized output in the basal ganglia–thalamo-cortical circuit and the ongoing abnormal modulation of motor programs by the cerebello-thalamo-cortical circuit, a conflict that could manifest as dyskinetic movements. Such a severe dysfunction of cerebellar sensory processing was demonstrated in advanced PD patients with LIDs (84, 86). Dyskinetic patients with LIDs had severe impairment of both homosynaptic and sensorimotor, heterosynaptic plasticity of M1. However, the sensorimotor M1 plasticity in PD could be temporarily reinstated even by a single session of inhibitory stimulation of cerebellum but not by sham stimulation, when patients were tested while on l-DOPA (84). Repeated sessions of cerebellar inhibitory stimulation had prolonged the antidyskinetic effect (84, 86) and were linked to the resurgence of sensorimotor M1 plasticity. Cerebellar cortical inhibition reinstated only sensorimotor M1 plasticity but not the intrinsic plasticity of M1. This suggested that only the unblocking of cerebellar sensory processing function by inhibition of the cerebellar cortex was involved in the resurgence of sensorimotor M1 plasticity and in the reduction of abnormal movements. Indeed, such inhibition of the cerebellar cortex was shown to reduce blood flow in the cerebellar cortex and dentate nucleus, in PD patients with LIDs (87). Based on these evidences it was proposed that the adjustment of the gain of cerebellar sensory processing is lost in advanced PD. The abnormal cerebello-dentato-thalamic outflow could lead to secondary maladaptive sensorimotor plasticity of M1 (84). It still remains to be elucidated whether the sensory processing dysfunction in PD is due to cerebellar cortical hyper-excitation triggered by the disease itself, as in the animal model of PD (76), or by an abnormal overdrive imposed by chronic non-physiological dopaminergic replacement. Testing the cerebellar plasticity in untreated or *de novo* PD patients may help resolving this conundrum.

## Conclusion and Future Directions

Recent studies establishing strong topography-specific connections among the basal ganglia, cerebellum, and their projections to overlapping areas in the motor cortices suggest that these networks influence each other’s functions. The contribution of the cerebello-thalamo-cortical pathway to tremor (88–90) and to LIDs in PD (84, 86) is now established. Whether there is a similar contribution of cerebellar processing function to other motor or cognitive symptoms in PD remains to be tested in future studies. There is preliminary evidence that DBS of subthalamic nucleus improves sensorimotor plasticity of M1 (91), but whether DBS also restores cerebellar processing function by re-instating normal signaling and plastic mechanisms in the cerebellar networks needs further exploration.

Both dopaminergic signaling in the basal ganglia and cerebellar sensory processing are necessary for scaling of M1 plasticity and topographic specificity (51). In *de novo* PD, sensorimotor plasticity is lost in the more affected hemisphere, while topographic specificity is lost in the less affected hemisphere with preservation of plasticity (68). This pattern suggests that loss of topographic specificity occurs prior to the loss of sensorimotor plasticity. These two defects could cause abnormal muscle synergies and thereby abnormal movements in PD. We propose that M1 plasticity, particularly associative sensorimotor plasticity, is an indication of motor map plasticity and therefore its loss or excess may have implications for motor learning and motor performance in disorders affecting the basal ganglia and cerebellum. Therapeutic interventions for such disorders might be more efficient if would attempt to normalize signaling in both striato-thalamo-cortical and cerebello-thalamo-cortical pathways.

## Conflict of Interest Statement

The authors declare that the research was conducted in the absence of any commercial or financial relationships that could be construed as a potential conflict of interest.
